# A Remarkable Case of Constipation

**Published:** 1887-11

**Authors:** J. B. Elliott

**Affiliations:** Brooklyn


### A REMARKABLE CASE OF CONSTIPATION.

J. B. ELLIOTT, M. D., BROOKLYN.

PREFACE.—A daily record for over thirty years of the name and prescription at office, or by visit of every patient attended, enables me to refer back for interesting or useful facts.

I am thus able to recall with considerable distinctness an experience of many years ago with a patient, the publication of which, though often designed, has long been delayed. Hoping it may prove of service to the timid or younger members of our school, who sometimes distrust natural powers with mild treatment under seeming great difficulties, it is now given with some minuteness as an event standing probably alone in the past and not likely to occur again in the future.

On the 21st of August, 1864, Mr. J., a young man of thin, sallow habit, about twenty years of age, was confined to his bed with a form of fever characterized by torpor and general prostration. A weak intellect and partial imbecility from birth no doubt favored this type of fever, and made him still more, even than in ordinary health, unsusceptible to impressions. I was called to the case soon after the attack, and learned from the stepmother, a very intelligent, kind-hearted lady of excellent family, the few particulars connected therewith.

The patient had drooped for some days, then became too ill to leave his bed, and gradually sank into a low, weak typhoid state, as the fever developed, in which he remained with very little change from day to day through (about) the usual period of this form of fever, in this instance a passive condition from beginning to end.

The man had no unusual tendency to inactive bowels in health. In this sickness they seemed merely to sympathize and join in the general torpor of the whole system while that continued. More than this, they were apparently the last part of the system to arouse when recovery had set in everywhere else.

This fact of improvement in general condition before the bowels naturally consent to move is a common observation in my experience when there has been brief interruption or unusual delay by sickness in other cases. Thus appetite and fresh supplies would seem important to discarding, yielding up, and throwing off the old.

In all cases of low vitality we fail to realize a ready response to remedies employed for restoration, however appropriate or wisely administered. But in this case, after more than a week had passed without an evacuation from the bowels, attention became directed particularly to this delay thereafter during the sickness. As time passed on without change in this respect more watchful attention was aroused and careful examinations made with reference to injury resulting to the patient from this cause. Daily watching found no tenderness, hardness, bloating, or tympanitis of the abdomen, kneading it gave no pain, pulse and temperature fair and unchanged, and general appearance of the patient satisfactory. Yet two and three weeks went by and no change. He grew no worse in any symptom, but became gaunt as a wolf. During the fourth week slight indications of improvement in pulse and tongue appeared, and by the end of it decided changes for the better in other respects occurred, particularly in a desire for food; still no movement of the bowels. In another week the patient was hungry for everything eatable. Instead of passively receiving the minimum quantity of liquid nourishment that had been ordered given with exactness every four hours from the beginning of the sickness he was eager for any kind of food, and solids as well as larger quantities were permitted by gradual increase from day to day as he continued to bear them, and yet no "action of the bowels." As he grew stronger daily, further addition to his regular meals was allowed, so that by the end of the sixth week he was eating as much as any laboring man would ordinarily require and wanted more. Where did all this food go? There was apparently little filling up of the still awful gaunt abdomen so persistent all through the sickness. Digestion and absorption must have acted vigorously, but certainly the bowels did not move. But confidence in the natural powers of the man, aided or unaided, certainly not impaired, by the mild remedies given, grew apace. Carefully watching effects, a still farther increase in food allowance was ordered. During the seventh week he ate enormously, but not all he wanted to. Our motto was, "Save some appetite for the next meal." Yet forty-eight days had brought no evacuation of the bowels. The patient was still gaunt, but evidently in good health.

He was digesting the food taken. Otherwise a lively internal rebellion must have resulted, as if from emetic, physic, or other poisonous material taken, and a forcible rejection of it followed. Inert, refuse material from which digestion and absorption has abstracted all fermentive or life force may remain a long time in the lower digestive tract without pain, harm, or disturbance to the patient. But in the case before us, when the forty-ninth day arrived with a healthy digestive action restored and stimulated by sufficient food, the farther natural result was ready to follow.

Entering the house on that day in my regular visit to the patient, the mother-nurse said she had something to show, and took me to the vessel, carefully set aside, in which, on removing the cover, we could observe the long-wished-for arrival—a first evacuation of the bowels in seven weeks. With the exception of the first dark, shriveled, hard portion, a more natural stool in shape and color could not have been asked for. The quantity was liberal, and as its expulsion was unattended with pain or violent effort, no shock or other evil effect followed to the patient. In due time his bowels became regular, as of old. He fully recovered, has remained well since, and is still residing in this city.

The mother often mentions him when we meet, and, with me, will never forget the event here related.

As the object of this paper was to relate facts with but little comment it might close here. A few words, however, as to the medical treatment of the case will be appropriate and, perhaps, expected. And yet there is little of importance to report on this score. In the law of similars many remedies are suited to constipation. But this condition is a natural one at the commencement of all fevers. The opposite state in them makes a bad complication. So the prescriptions in this case referred to general symptoms, of which constipation was only one element, although that became more conspicuous afterward by persistent continuance beyond all precedent as a fact, yet not as a disturbing cause to the patient. Later on such remedies as Plumbum, Opium, Alumina, and other similar agents for constipation alone were employed, but with no more prompt response thereto than to other specifics given. Torpor, without responsive reaction to anything, was the rule. Some may ask, "Why not have used a simple enema in the case?" Educated and experienced during early years in this and the old cathartic plans, if any better result was to be expected from that source I ought to have been aware of it. But my patient was doing well. Probably a fever of this form never went through its course more quietly, with less pain, in a shorter period of time, convalesced more rapidly, or made a more perfect recovery than did this one. A simple enema or mild cathartic could and probably would painfully bloat or gripe, and a more violent drastic poison act worse or fatally upon the patient. At best a forced evacuation of the bowels only could result from their use, which doubtless would have made him uncomfortable and retarded rather than promoted recovery.

From close observation as to effects of retarded or interrupted movements of the bowels so common and natural in sickness, I am satisfied no evil results follow from the same, either immediate or remote, unless violent means have been employed to force the evacuation. A mild remedy designed to simply excite natural action on the law of similars can safely be trusted in such cases. The kidneys are the grand laboratory for eliminating and sending off poisonous material from the system. They must and therefore do act constantly in this duty. When they become diseased and cease to perform their natural functions, blood-poisoning, indicated by brain and other general disturbances, is an immediate result. Not so with the alimentary canal, which can remain so long inactive without injurious consequences. In such an event it is not unlikely the kidneys assume a double duty for the time, and so far as necessary remove deleterious elements that might otherwise pass off through the channel first named.

